# Choreography of Lyme Disease Spirochete Adhesins To Promote Vascular Escape

**DOI:** 10.1128/spectrum.01254-23

**Published:** 2023-05-31

**Authors:** Xi Tan, Mildred Castellanos, George Chaconas

**Affiliations:** a Department of Biochemistry & Molecular Biology, University of Calgary, Calgary, Alberta, Canada; b Department of Microbiology, Immunology & Infectious Diseases, Snyder Institute for Chronic Diseases, University of Calgary, Calgary, Alberta, Canada; Institute of Parasitology, Biology Centre, ASCR

**Keywords:** Lyme disease, adhesins, extravasation, hematogenous dissemination, intravital imaging

## Abstract

The Lyme disease spirochete Borrelia burgdorferi *sensu lato* can cause a multitude of clinical manifestations because of its ability to disseminate into any organ system via migration through soft tissue, the lymphatic system, and the circulatory system. The latter is believed to constitute the predominant pathway for dissemination to distal sites from the inoculating tick bite. In spite of its importance, the hematogenous dissemination process remains largely uncharacterized, particularly due to difficulties studying this process in a living host and the lack of an *in vitro* system that recapitulates animal infection. In the current work, we provide the first information regarding the stage of the vascular transmigration pathway where three important adhesins function during invasion of mouse knee joint peripheral tissue from postcapillary venules. Using intravital imaging coupled with genetic experiments employing sequential double infection, we show a complex temporal choreography of P66, decorin binding proteins (DbpA/B), and outer surface protein C (OspC) at discrete steps along the pathway of vascular escape, underscoring the importance of B. burgdorferi adhesins in hematogenous dissemination in the mouse knee joint and the complexity of vascular transmigration by a disseminating pathogen.

**IMPORTANCE** Lyme disease is caused by the spirochete Borrelia burgdorferi, which is transmitted by a bite from an infected tick. Disease development involves a complex series of host-pathogen interactions as well as dissemination of the infecting organisms to sites distal to the original tick bite. The predominant pathway for this is believed to be hematogenous dissemination. The mechanism by which the spirochetes escape circulation is unknown. Here, using intravital microscopy, where the Lyme spirochete can be observed in a living mouse, we have studied the stage in the vascular escape process where each of three surface adhesins functions to facilitate escape of the spirochete from postcapillary venules to invade mouse knee joint peripheral tissue. A complex pattern of involvement at various locations in the multistage process is described using a unique experimental approach that is applicable to other disseminating pathogens.

## INTRODUCTION

Lyme disease is a tick-transmitted illness that is increasing in incidence and is a growing concern throughout the northern hemisphere (1–4). The disease is unusual in its ability to promote a wide variety of clinical manifestations resulting from the ability of the causative agent, the spirochete Borrelia burgdorferi, to invade virtually all organ systems. Although the spirochete can disseminate through soft tissue (5–7) and the lymphatic system (8–10), the primary route of dissemination to locations distal from the original tick bite is believed to be by travel through the bloodstream. Yet little is known regarding the vascular transmigration process whereby B. burgdorferi escapes the circulatory system to invade adjacent tissue. This is due to the small number of spirochetes extravasating at any given time coupled with the difficulties involved in studying a dynamic process such as this within an animal and the lack of an *in vitro* system that mirrors vascular escape in a vertebrate host.

To study the hematogenous dissemination process, we have made use of intravital microscopy to visualize the dynamic behavior of spirochetes in a living mouse (11, 12). Our recent work has shown that the vascular transmigration process for B. burgdorferi into the mouse knee joint peripheral tissue involves several stages. An early step is interaction of the spirochetes with the vascular endothelium (12–14). This is followed by induction of endothelial activation (15), which we have assessed by observation of neutrophil recruitment and by the induction of vascular permeability (16). Yet, although activation of the endothelium in postcapillary venules is necessary for spirochete extravasation, it is not sufficient. An additional step, which we have termed potentiation, is required to prepare the endothelium for vascular transmigration of B. burgdorferi. Potentiation is assessed by simply monitoring whether spirochete extravasation can occur (16). The nature of the changes that comprise potentiation remains unknown at this time; however, potentiation can be induced by several cytokines whose serum levels increase after infection (interleukin 10 [IL-10], monocyte chemoattractant protein 1 [MCP-1], and tumor necrosis factor alpha [TNF-α]), but not others (IL-1β, IL-4, granulocyte-macrophage colony-stimulating factor [GM-CSF], and IL-17) that nonetheless promote endothelial activation (16). Potentiation requires about 24 h after infection to occur and readies the endothelium to allow spirochete extravasation via a transcellular pathway in the mouse knee joint (16).

During our studies of the vascular transmigration process, we have looked for a possible role for spirochete adhesins in the process. Adhesins are multifunctional proteins and are a touchpoint for interaction of B. burgdorferi with the host, potentially promoting binding to various accessible host components such as the extracellular matrix (ECM), as well as inducing signaling in host cells (1, 17–20). Our earlier studies have shown a role for the fibronectin and glycosaminoglycan (GAG) binding adhesin BBK32 (13, 14) and the dermatan sulfate binding adhesin VlsE in early vascular interactions (21). In this work, we focus on the role in vascular transmigration of three important B. burgdorferi adhesins, P66, outer surface protein C (OspC), and decorin binding proteins A and B (DbpA/B). P66 is a porin (22–25) and integrin binding protein (26, 27) that plays a role in *in vivo* survival and efficient dissemination (28, 29). The integrin binding activity is required for extravasation into knee joint peripheral tissue (30). OspC binds fibronectin and/or dermatan sulfate GAGs (31), plasminogen (32), complement factor C4b (33), fibrinogen (34), and a tick salivary protein that inhibits complement (35, 36). A rationally designed OspC mutant unable to bind to fibronectin or dermatan sulfate is unable to promote vascular transmigration into knee joint peripheral tissue (31). DbpA and its paralog, DbpB, bind to decorin, an ECM component (37, 38). Reduced virulence and dissemination result in the early stages of infection from a loss of either paralog (8, 39–41). DbpA/B have been reported to influence tissue tropism (42–44). The phenotypes of mutations in P66, OspC, and DbpA/B are quite variable depending upon infection conditions, mouse strains, and assay methods, and the reader is referred to several reviews for further details and a comprehensive literature review (1, 17–20).

In the current work, we use intravital imaging coupled with spirochete genetics and sequential double-infection experiments to show a complex temporal choreography of DbpA/B, OspC, and P66 at discrete steps along the multistep pathway of vascular escape, underscoring the importance of these three B. burgdorferi adhesins in hematogenous dissemination into the knee joint peripheral tissue in *Cd1d^−/−^* mice.

## RESULTS

### DbpA/B are required for vascular transmigration into knee joint peripheral tissue.

**FIG 1:**
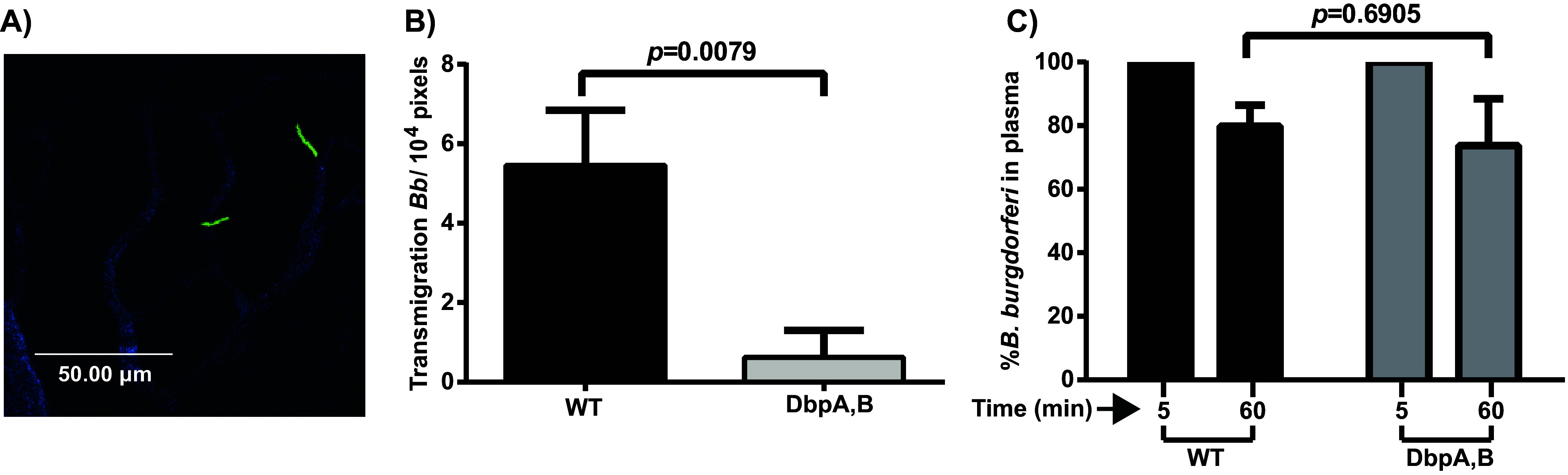
Effect of DbpA/B on vascular transmigration and clearance of B. burgdorferi-infected mice. Mice were infected with either GFP-expressing wild-type B. burgdorferi (GCB4446; see Table S1 in the supplemental material) or B. burgdorferi carrying a well-characterized *dbpA/B* deletion (41) in GCB4032 (Table S1). Twenty-four hours after B. burgdorferi infection, vascular transmigration was scored in the knee joint-proximal tissue (*n* = 5). (A) Twenty-four hours after GFP-labeled B. burgdorferi infection, vascular transmigration was scored in the knee joint-proximal tissue (*n* = 5) by intravital imaging. Blood vessels were stained with Alexa Fluor 647-conjugated anti-mouse CD31 (PECAM-1) antibody shown in blue. Scale bar, 50 μm. (B) The data are plotted as the number of transmigrated spirochetes observed in an area of 10^4^ pixels. Statistical significance was analyzed using the nonparametric Mann-Whitney test. (C) Clearance of B. burgdorferi from the vasculature in the mice used in panel A was monitored by withdrawal of blood through the tail vein at 5 and 60 min postinoculation. Plasma was recovered, and spirochetes in the plasma were directly counted by dark-field microscopy. The data are plotted for each mouse as the percentage of spirochetes in plasma at 60 min relative to the 5-min time point, which was defined as 100% for each mouse.

Our experimental system to study vascular escape uses intravital microscopy to image green fluorescent protein (GFP)-expressing B. burgdorferi in postcapillary venules (Fig. 1A) in the knee joint peripheral tissue following intravenous (i.v.) injection of *Cd1d^−/−^* mice (30). *Cd1d^−/−^* mice are necessary for these experiments because they lack invariant natural killer T (iNKT) cells, which are the primary line of defense in the knee joint and disrupt B. burgdorferi dissemination into the joint (45, 46). We have previously described a requirement for two B. burgdorferi surface adhesins for vascular escape of the spirochetes into the mouse joint peripheral tissue, the porin and integrin- binding protein P66 (30) and the outer surface protein OspC (31). We now report the involvement of a third adhesin, the decorin binding protein family members DbpA/B (47). DbpA is allelically variable and has been shown to confer tissue tropism, including invasion of the mouse joint (42). Use of a B. burgdorferi strain carrying a well-characterized *dbpA/B* deletion (41) resulted in a decrease in vascular transmigration through postcapillary venules in joint peripheral tissue to a value of 11% of the wild-type (WT) level (Fig. 1B). A control was also performed to assess spirochete clearance from the vasculature in the absence of DbpA/B, which would perturb the measured level of spirochete extravasation in our intravital assay. No change in the rate of clearance was observed in the absence of DbpA/B from the surface of the spirochete (Fig. 1C).

### Probing a role for B. burgdorferi adhesins in endothelial activation: P66, but not DbpA, DbpB, or OspC, plays a major role in endothelial activation.

**FIG 2:**
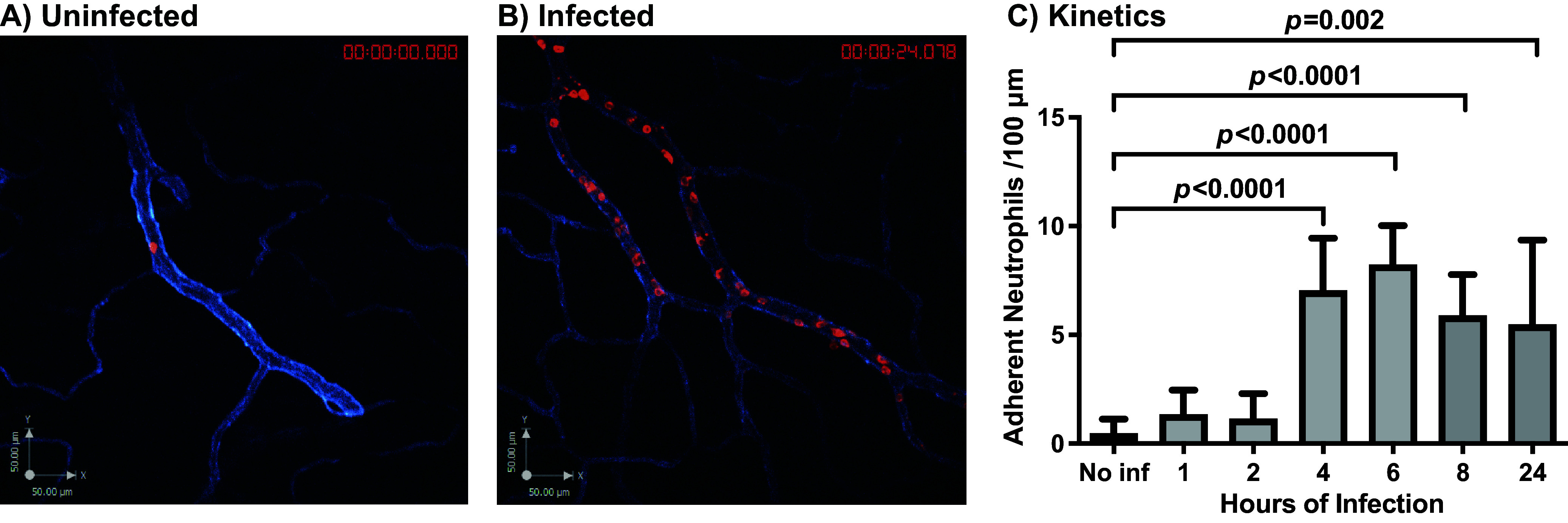
Neutrophil adhesion to monitor endothelial activation in the knee joint-proximal tissue of B. burgdorferi*-*infected mice. Intravital imaging was performed in uninfected (A) and B. burgdorferi (GCB726)-infected (B) mice 24 h postinfection. Neutrophils were stained with anti-Ly6G (red), and the joint-proximal venules were stained with anti-PECAM-1 (blue). (C) *Cd1d^−/−^* mice were infected with wild-type GFP-expressing B. burgdorferi (GCB726) for the indicated times (*n* = 3 mice/group, 3 × 10^8^ spirochetes per mouse). Uninfected mice injected with PBS were used as a negative control. Neutrophil adhesion to the endothelium was assessed by intravital imaging as noted in Materials and Methods and as previously described (16). Statistical significance was analyzed using the Kruskal-Wallis test. Error bars indicate SD in all plots.

We have previously reported that intravenous injection of B. burgdorferi into *Cd1d^−/−^* mice resulted in endothelial activation in postcapillary venules followed by potentiation of the endothelium for spirochete extravasation into joint peripheral tissue at 24 h postinfection (16). Here, we further analyze this course of events beginning with a kinetic profile of endothelial activation in living mice as assessed by neutrophil adherence using intravital microscopy. Figure 2A and B shows anti-Ly6G (red)-stained neutrophils in an uninfected and infected mouse postcapillary venule, respectively (see also Video S1 and S2 in the supplemental material). Figure 2C shows a kinetic profile of endothelial activation following B. burgdorferi injection. We observed an onset of endothelial activation at 4 h postinfection with persistence through 24 h, at which time extravasated spirochetes are visible surrounding the knee microvasculature (16).

**FIG 3:**
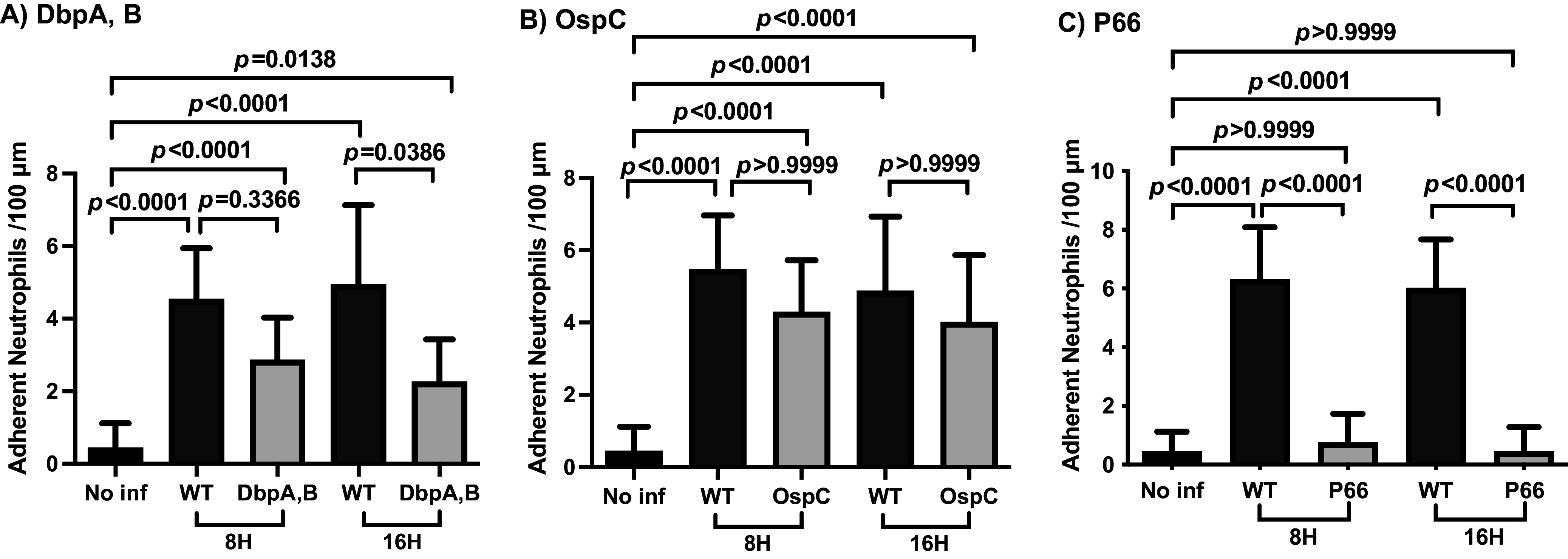
Neutrophil adhesion as a measure of endothelial activation in the knee joint-proximal tissue of B. burgdorferi mutant-infected mice. (A) *Cd1d^−/−^* mice were infected with GFP-expressing wild-type B. burgdorferi (GCB4446) or a DbpA/B mutant strain (GCB4032) for 8 and 16 h (*n* = 3 mice/group, 3 × 10^8^ spirochetes per mouse). Uninfected control mice were injected with PBS. Neutrophil adhesion was assessed as described in Materials and Methods. (B) *Cd1d^−/−^* mice were infected with GFP-expressing wild-type B. burgdorferi (GCB4458) or the OspC_ECM_ mutant strain (GCB4452) for 8 and 16 h (*n* = 3 mice/group, 3 × 10^8^ spirochetes per mouse). Neutrophil adhesion was assessed. (C) *Cd1d^−/−^* mice were infected with GFP-expressing wild-type B. burgdorferi (GCB847) or a P66-integrin binding mutant (P66_D205A,D207A,_ GCB3003) for 8 and 16 h (*n* = 3 mice/group, 3 × 10^8^ spirochetes per mouse). Neutrophil adhesion was assessed as described above.

Because endothelial activation and potentiation are requirements for B. burgdorferi vascular escape (16), we probed the possible role in endothelial activation of each of the three adhesins needed for extravasation by quantifying neutrophil adherence to the endothelium at 8 and 16 h after spirochete injection. As shown in Fig. 3A, the absence of DbpA/B (which block spirochete extravasation) resulted in a less than 2-fold decrease in neutrophil adherence that was not statistically significant at 8 h. At 16 h, a similar but significant decrease was observed. These results suggest that DbpA/B might play a small, nonessential role in endothelial activation; however, the magnitude of the impact on neutrophil adherence is much smaller than on spirochete transmigration (Fig. 1), suggesting a predominant function at an alternate step in the transmigration pathway.

The same experimental design using spirochetes carrying the OspC_ECM_ mutation (31), which can neither bind the ECM nor extravasate, resulted in a slight and statistically insignificant decrease in neutrophil adhesion at both time points (Fig. 3B), suggesting that the predominant role of OspC is not to activate the endothelium. Finally, B. burgdorferi harboring the P66_D205A,D207A_ mutation in the integrin binding residues (29, 30) displayed a major deficit in promoting neutrophil adhesion to the vasculature at both time points (7% of wild-type levels at 16 h) and appears to be unable to induce endothelial activation.

To further probe the importance of P66 in preparing the endothelium for spirochete transmigration, we used an alternative approach. Since several cytokines are synthesized after B. burgdorferi infection, primarily by neutrophils, and several can potentiate the endothelium for B. burgdorferi extravasation (16), we assessed the level of 17 cytokines in mouse plasma at 24 h postinoculation (Fig. S1), comparing cytokine levels in uninfected mice versus mice infected with WT B. burgdorferi and spirochetes carrying the P66 integrin binding mutant, P66_D205A,D207A_. Eight cytokines were either not induced by WT B. burgdorferi or did not show a decrease in levels between the WT and the P66_D205A,D207A_ mutant (Fig. S1B). In contrast, nine cytokines displayed significant increases following infection by WT spirochetes and displayed decreases relative to WT by the P66_D205A,D207A_ mutant (Fig. S1A). Among the cytokines whose expression was affected by the P66 mutant were the potentiation-inducing cytokines IL-10, MCP-1, and TNF-α. The sum of the results in Fig. 3 and Fig. S1 indicates that the integrin binding residues of P66 play an important role in endothelial activation and the expression of several cytokines following B. burgdorferi infection.

### Bypass of endothelial activation and potentiation reveals that P66 and OspC, but not DbpA/B, play a role in extravasation.

**FIG 4:**
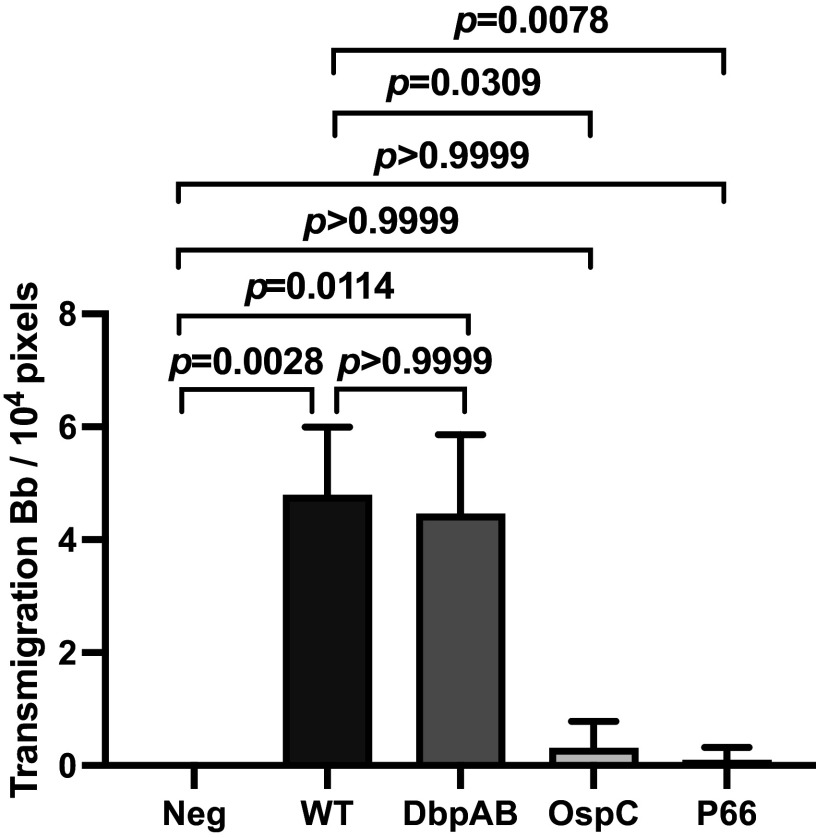
Intravital imaging to investigate the effect of MCP-1 on vascular transmigration of B. burgdorferi-infected mice. *Cd1d^−/−^* mice received PBS (Neg) or 5 μg/25-g mouse of MCP-1 by i.v. injection 1 h prior to injection of a GFP-expressing B. burgdorferi strain. WT, GCB847; ΔDbpA/B, GCB4032; OspC_ECM_, GCB4452; P66_D205A,D207A_, GCB3003. Mice were infected with the above-described strains 3 h before imaging (*n* = 5 mice/group, 3 × 10^8^ spirochetes per mouse). The total number of the GFP-expressing spirochetes outside the vasculature was counted; the data are plotted as the number of transmigrated spirochetes observed in an area of 10^4^ pixels (see Fig. 1 and Materials and Methods). Statistical significance was analyzed using the Kruskal-Wallis test. Error bars indicate SD in all plots.

As a means of bypassing the endothelial activation and potentiation requirement for B. burgdorferi to transmigrate the endothelium, these processes can be promoted by injection of mice with one of several cytokines (IL-10, MCP-1, or TNF-α), which can rapidly prepare the endothelium for spirochete extravasation within 3 h of infection, rather than the 24-h time period required during a normal infection (16). To determine whether DbpA/B, OspC, and P66 play a role in vascular escape after potentiation has occurred, we performed the experiment shown in Fig. 4. Mice were injected with MCP-1 1 h prior to infection with the indicated B. burgdorferi WT or mutant strains. Intravital imaging was performed 3 h after infection to monitor vascular transmigration of spirochetes (16). At the 3-h time point, transmigration of WT B. burgdorferi did not occur in untreated mice (Fig. 4, negative control) (see our previous work [16]). However, as we previously observed, MCP-1-treated mice displayed robust levels of WT spirochete transmigration at 3 h. Interestingly, the DbpA/B deletion strain, which is severely compromised for transmigration in untreated mice (see Fig. 1), showed WT levels of transmigration (Fig. 4). The results from this experiment suggest that after endothelial activation and potentiation, DbpA/B are not required for the extravasation process. The apparently limited role of DbpA/B in activation suggests that the severe defect of the DbpA/B deletion strain is due to a predominant role of DbpA/B in potentiation.

In contrast, both OspC (which plays no role in activation) and P66 (which is essential for activation) appear to play a role in the vascular escape process subsequent to potentiation since MCP-1, which potentiated the endothelium, cannot rescue them (Fig. 4).

### Sequential double-infection experiments reveal that DbpA/B, OspC, and P66 play roles at discrete phases of the vascular transmigration process.

**FIG 5:**
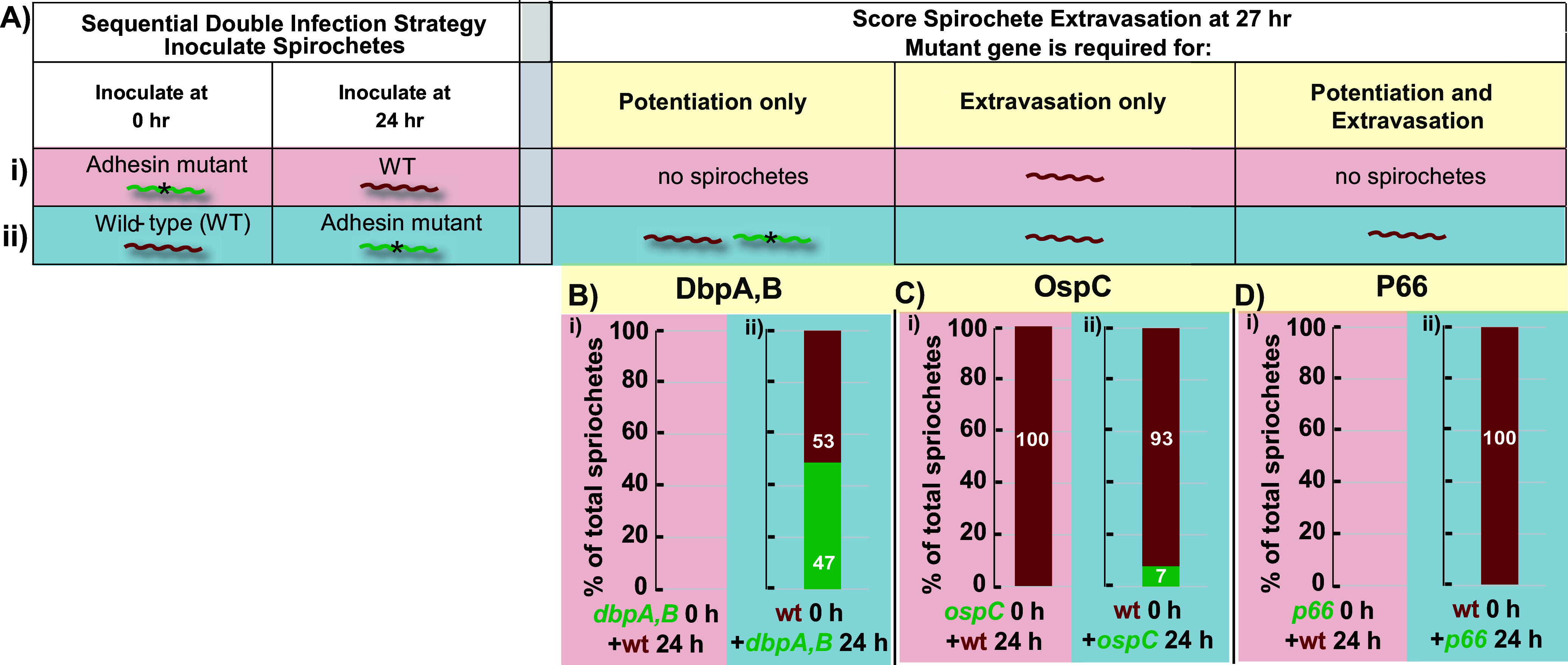
Sequential double-infection experiment using three adhesin mutants. (A) Possible results of the sequential double-infection intravital microscopy (IVM) transmigration experiment. (B to D) The bar graphs represent the relative percentage of *dbpA/B*, *ospC*, or *p66* mutant and wild-type spirochete strains observed outside the vasculature in the double infections. Double-infection strategies are as follows: (i) adhesin mutant *dbpA/B*, *ospC*, or *p66* (GFP) at 0 h→wild type (Tomato) at 24 h and imaging at 27 h, or (ii) reverse infection-order wild type (Tomato) at 0 h→adhesin mutants *dbpA/B*, *ospC*, or *p66* (GFP) at 24 h and imaging at 27 h; *n* = 4 for all groups. The total numbers of the GFP- and Tomato-expressing spirochetes outside the vasculature were counted (Fig. S2 in the supplemental material contains the raw data).

Potentiation requires extended, e.g., 24-h, infection, whereas extravasation across (previously) potentiated endothelium occurs within 3 h. This temporal distinction permits us to determine whether an adhesin functions during the (early) potentiation phase or the (late) extravasation phase by inoculating mice with an adhesin mutant and a wild-type strain in sequence. In fact, we utilize two types of sequential double-infection experiments (16). In experiment i (shown in pink in Fig. 5A), a GFP-expressing adhesin mutant (green) was injected at time point 0 followed by injection of Tomato-expressing WT (red) spirochetes at 24 h. Three hours later, intravital microscopy was performed to score green and red spirochetes that had extravasated. The columns on the right show the predicted results. If the mutation lies in an adhesin that plays a role in extravasation only, the endothelium will be potentiated by the mutant spirochetes, resulting in vascular escape by the red WT (but not green mutant) B. burgdorferi. If the adhesin carrying the mutation plays a role in potentiation alone, or both potentiation and extravasation, then the endothelium cannot be potentiated, and neither green nor red spirochetes will be observed.

The ambiguity of the latter result can be resolved with experiment ii, a second type of sequential double-infection experiment (shown in blue in Fig. 5A), in which the order of injection is reversed, i.e., the WT red spirochetes are injected at time point 0 followed by the green adhesin mutants at 24 h. If the mutated adhesin is involved exclusively in potentiation, then the WT spirochetes will induce potentiation, and both green mutant and red WT spirochetes will be observed at 27 h. Alternatively, if the mutated adhesin is involved exclusively in extravasation, then only the red WT spirochetes will escape the vasculature. Finally, if the mutated adhesin is involved in both potentiation and extravasation, then again, only red WT spirochetes will be observed outside the vasculature. By combining both types (i and ii) of sequential double-infection experiments, the role of an adhesin in potentiation and/or extravasation can be unambiguously discerned (Fig. 5A).

We utilized this method to identify the stages in vascular transmigration at which DbpA/B, OspC, and P66 are active. The results from experiments i (pink) and ii (blue) are shown in Fig. 5B to D, with the raw data plotted in Fig. S2. Remarkably, each adhesin displayed a unique functional profile: DbpA/B are involved in potentiation, OspC in extravasation, and P66 in both potentiation and extravasation. A point worthy of note is that due to possible redundancy of adhesin functionality, our experiments may not reveal all functional aspects of particular adhesins if other adhesins can pitch-hit for them in their absence. Nonetheless, the conclusions from the sequential double-infection experiments are in complete agreement with those from Fig. 3 (measuring endothelial activation) and Fig. 4 (measuring transmigration after artificial potentiation) using alternative experimental approaches that provide additional functional information for the investigated adhesins.

## DISCUSSION

Hematogenous dissemination is a prominent pathway for B. burgdorferi spirochetes to travel to distal sites from the inoculating tick bite and escape from postcapillary venules to invade the surrounding tissues. The vascular escape process has remained mechanistically uncharacterized and is difficult to study. Here, we describe several types of experiments using intravital microscopy to study this process in living mice. The experimental system that we employ is, by necessity, not fully reflective of a natural infection because of (i) the high dose of spirochetes that we inject directly into the mouse required to image them; and (ii) the *Cd1d^−/−^* mice that lack iNKT cells, which would otherwise disrupt dissemination and phagocytize extravasated spirochetes in the knee joint (46, 48). In addition, extrapolation of our results in the knee joint microvasculature to other dissemination sites requires experimental testing due to the existence of organotypic vasculature (49), which may impose a requirement for different pathogen proteins for extravasation at different sites. Nonetheless, our model is the only experimental system in which to study the mechanistic properties of spirochete transmigration during animal infection and has resulted in unique insights into the tissue colonization process by B. burgdorferi (11–14, 16, 21, 31, 46, 48), including the demonstration of a role for the adhesins P66 and OspC in vascular transmigration and the discovery that distinct stages of the extravasation process could be temporally separated.

**FIG 6:**
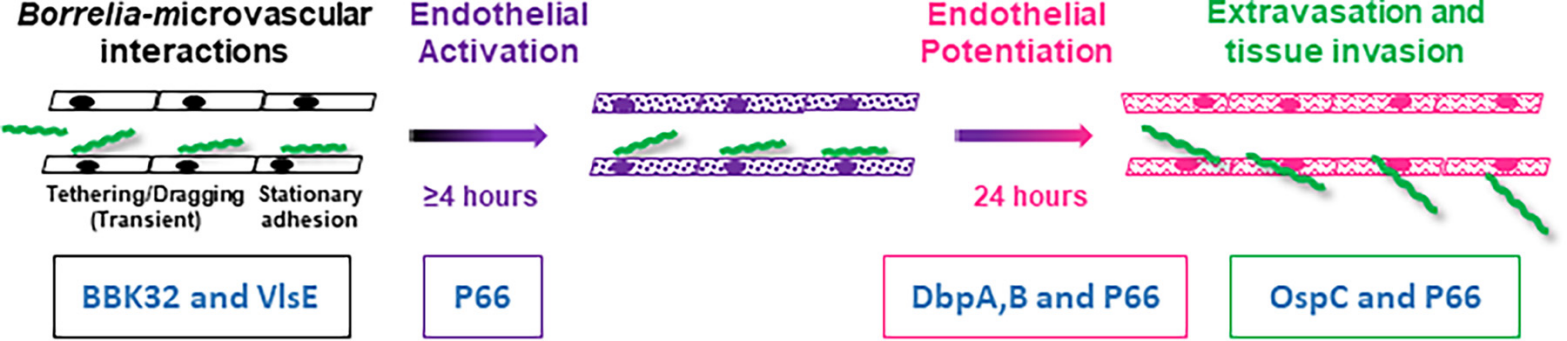
Summary of stages at which various B. burgdorferi adhesins play a role in microvascular interactions and vascular transmigration in knee joint peripheral tissue in *Cd1d^−/−^* mice. The steps at which five B. burgdorferi adhesins function are shown. The adhesins BBK32 (13, 14, 57, 58) and VlsE (21) contain potent adhesive properties that mediate early interactions between the vascular endothelium and B. burgdorferi spirochetes. Subsequently, three adhesins whose functional absence blocks vascular escape (P66 [30], DbpA/B [see Fig. 1], and OspC [31]) mediate different aspects of vascular transmigration at the steps shown above.

Here, the collective results lead, for the first time, to the assignment of B. burgdorferi adhesin function to discrete steps in vascular transmigration shown in the scenario in Fig. 6. The adhesins P66, DbpA/B, and OspC are each required for vascular escape, but each makes contributions at different stages of the process. P66 is a membrane protein with porin (22) and integrin binding activity (26). The integrin binding activity is required for vascular escape of B. burgdorferi from postcapillary venules in the knee joint microvasculature (30). We have now mapped a role for this multifunctional protein at three discrete steps in vascular transmigration: in endothelial activation (Fig. 3), endothelial potentiation (Fig. 5) as well as the extravasation step (Fig. 5). Endothelial activation is likely an indirect effect through induction of cytokine expression by neutrophils (16), while potentiation may be direct or indirect. All three activities are dependent upon P66 integrin-binding activity, and it is tempting to speculate that endothelial activation and potentiation may result from integrin signaling. This idea is supported by the ability of P66 to affect endothelial gene expression in cultured cells (50). The mechanism by which P66 is involved in the extravasation step also remains mysterious at this time and may be direct or indirect. The molecular details of the tripartite activities of P66 in vascular transmigration remain to be elucidated; however, they do not appear to be related to vascular adhesion under shear force, as P66 is unable to promote such vascular interactions (30).

The second adhesin that we have identified (31) as required for vascular transmigration out of the knee joint microvasculature is OspC (51). This protein binds to the ECM, recognizing GAGs or fibronectin, in a variant-dependent manner (31). Unlike P66 and DbpA/B, OspC does not appear to play a role in endothelial potentiation but instead functions at the extravasation step and requires its ECM binding residues to do so (Fig. 5). Whether the requirement for OspC in extravasation is direct or indirect is currently unknown.

The third adhesin that we have identified as required for escape from the knee joint microvasculature is the decorin binding paralogs DbpA/B (52) (Fig. 1). The data presented here suggest that their activity in vascular transmigration is predominantly at the endothelial potentiation stage (Fig. 5). Although mutants of DbpA that are defective in decorin binding exist (53), construction of strains harboring these mutations in the GFP-expressing strain required for intravital imaging has not been successful, although several attempts have been made; an assessment of the decorin binding residues in potentiation remains a future goal.

While mechanistic information is lacking on how the various adhesins studied here promote vascular transmigration, a recent paper on DbpA/B has reported that either of these proteins can promote enhanced motility of B. burgdorferi spirochetes in the ECM, suggesting a role for these paralogs in dissemination (54). Based upon these findings, one might have expected that the function of DbpA/B would also have been mapped to the extravasation step of the transmigration pathway (Fig. 6). However, if either P66, OspC, or some as-yet-unidentified adhesion could substitute for DbpA/B in its absence, then our experiments would be unable to map such a function. Another important finding on DbpA-decorin interactions is the increased stability of these interactions when force loaded to facilitate the ability of the spirochetes to withstand shear stress (54). This property is expected to stabilize interactions of adhesins with the microvasculature at all stages (endothelial activation, potentiation, and extravasation) of the vascular transmigration pathway (Fig. 6). These recently reported properties for DbpA should also be investigated for P66 and OspC, as they provide mechanistic information on how adhesin-ligand interactions may function in the vascular transmigration pathway.

In summary, the work described here opens a new door for further investigation of the vascular transmigration process of the Lyme disease spirochete B. burgdorferi and the role played by various adhesins. P66, DbpA/B, and OspC work together to both promote changes in the host endothelium and then capitalize on those changes to extricate the pathogenic spirochetes from the microvasculature and into the surrounding tissue. Our experimental approaches using an intravital imaging readout provide the first inroads to dividing the vascular escape process into various stages and to determine at which stages three adhesins, each of which is essential for vascular transmigration, function. There may be additional stages at which each adhesin functions that could not be detected if there is functional redundancy resulting in complementation when an adhesin is absent. This powerful experimental system is particularly valuable for studies on vascular escape of the Lyme disease spirochete and other pathogens where no cell culture system that recapitulates a mammalian infection exists. In the current work, we use intravital imaging coupled with spirochete genetics and sequential double-infection experiments to show a complex temporal choreography of P66, DbpA/B, and OspC at discrete steps along the pathway of vascular escape, underscoring the importance of B. burgdorferi adhesins in hematogenous dissemination. Future studies will focus on the molecular mechanisms by which these adhesins facilitate vascular escape of the Lyme disease spirochete.

## MATERIALS AND METHODS

### Ethics statement and use of mice.

All animal experimentation was carried out in accordance with the principles outlined in the most recent policies and *Guide to the Care and Use of Experimental Animals* by the Canadian Council on Animal Care. The animal protocols (AC16-0244 and AC20-0159) were approved by The Animal Care Committee of the University of Calgary. Wild-type BALB/c mice were purchased from Charles River Laboratories (Wilmington, MA), and *Cd1d^−/−^* mice in a BALB/c background (Jackson Laboratories; no. 2962) were bred in-house at the Clara Christie Centre for Mouse Genomics at the University of Calgary. Mice of both genders between 6 to 8 weeks of age were used.

### Antibodies and reagents.

Alexa Fluor 647-conjugated anti-mouse CD31 (PECAM-1; clone 390), phycoerythrin (PE)-conjugated anti-mouse Ly6G antibody (clone 1A8), and carrier-free mouse cytokine MCP-1 (catalog no. 578404) were from BioLegend Inc. (San Diego, CA).

### Bacterial strains and cultures.

The B. burgdorferi strains used in this study are described in Table S1 in the supplemental material. Spirochete cultures in Barbour-Stoenner-Kelly II (BSK-II) medium were inoculated from frozen glycerol stocks. The spirochete cultures were grown at 35°C to a concentration of around 5 × 10^7^/mL in 50 mL BSK-II medium. Spirochetes in 50 mL were harvested by centrifugation at 6,000 × *g* for 15 min at 4°C and washed twice with 100 mL of cold phosphate-buffered saline (PBS) as previously described (30) and resuspended to a density of 2 × 10^9^/mL spirochetes in cold PBS for mouse infection. The Escherichia coli strains were cultivated at 37°C in Luria-Bertani broth or agar (BD Bioscience, Franklin Lakes, NJ), with ampicillin (100 μg/mL) or no antibiotics when appropriate.

### Strain construction.

The B. burgdorferi B31 5A4 *dbpA/B* knockout (KO) strain (GCB4433; see Table S1) was obtained by transforming GCB2958 with 47 μg of the plasmid pNP3 (41), which harbors the *dbpA/B* region interrupted by the *gent* gene expressed by the *flgB* promoter. Gentamycin-resistant transformants were screened for the presence of the *gent* gene with oligonucleotides B348 and B349 (55). We synthesized oligonucleotides B2914 (ATACGAGAGTCCACTTTATTTGC), B2915 (AAGTCTTCTGCACCAAGA), B2916 (GTAAGACATTCATCCGCT), and B2917 (GCATAGCAAGCTTGTAATTCC) to amplify the junctions *dbpA-gent* and *gent-dbpB* to confirm the presence of the mutation. Oligonucleotide pair B2914 and B2916 produced a 768-bp band, while the pair B2915 and B2917 amplified a band of 537 bp. Oligonucleotides B2914 and B2917 amplified a 1.7-kb band expected when the *gent* cassette was inserted. PCRs were performed with Phusion polymerase (New England Biolabs) following the manufacturer’s instructions. Finally, analysis to confirm genomic plasmid content was performed using multiplex PCR as previously described (56) for this and all other strains constructed.

To generate the fluorescent B31 5A4 *dbpA/B* KO strain (GCB4032), we transformed GCB4433 with 55 μg of DNA from the previously described pTM61*kan*, *gfp* (12, 21). Transformants were selected as kanamycin resistant and screened for GFP fluorescence and for the presence of the *kan* cassette with oligonucleotides B70 and B71 (55).

To generate fluorescent B. burgdorferi 5A4 carrying pTM61*kan*, *gfp* (GCB4446), we transformed GCB2958 with 75 μg of methylated DNA from the previously described pTM61*kan* (21). Methylation was performed by using SssI methyltransferase from New England Biolabs. Reactions were cleaned with phenol-chloroform, dialyzed, and concentrated to a volume of 10 μL. Transformants were selected for kanamycin resistance and were screened for GFP fluorescence and the presence of the *kan* cassette with oligonucleotides B70 and B71 (55).

### Mouse infection.

BALB/c or *Cd1d*^−/−^ mice were anesthetized by intraperitoneal injection of 200 mg/kg ketamine hydrochloride (Bimeda-MTC Animal Health Inc., Cambridge, ON) and 10 mg/kg of xylazine hydrochloride (Bayer Inc., Toronto, ON). For vascular transmigration and adhesion assays, anesthetized *Cd1d*^−/−^ mice were secured in a mouse restrainer and inoculated by tail vein injection with 3 × 10^8^ spirochetes in 150 μL of PBS.

### Intravital imaging.

**(i) Vascular transmigration assay.** Details of the vascular transmigration assay for B. burgdorferi intravitally were as previously described (16, 30, 31). The surgically prepared knee joint region was typically about 220 mm^2^ (20,646 pixels). The observed area was kept under a glass coverslip (VWR Scientific; catalog number 48393-026; 22 by 30 mm, 660 mm^2^, 61,744 pixels) that also served as a size standard, allowing the determination of the imaging area surrounding the knee joint by using Photoshop. To assess B. burgdorferi transmigration, all available fields of view (hundreds) were imaged, and the total number of fluorescent spirochetes outside the vasculature (stained with PECAM-1 antibody) was counted. Because the imaging area varies for individual mice, the total number of escaped spirochetes was divided by the size of the area imaged for each mouse, and the data were plotted as the number of escaped spirochetes in an area of 10^4^ pixels for each mouse.

**(ii) Spirochete clearance assay.** The details of the spirochete clearance assay have been previously reported (16). Basically, 35 μL of blood was recovered from the tail vein and processed as described (16). Spirochetes were counted using a Petroff-Hausser counting chamber using dark-field microscopy. Because the absolute concentration of spirochetes in each mouse was variable, the data were plotted as the percentage of spirochetes in plasma at 60 min relative to the 5-min time point, which was defined as 100% for each mouse. The absolute concentration of spirochetes was roughly 10^7^ spirochetes/mL at the 5-min time point.

**(iii) Neutrophil recruitment assay.** Intravital microscopy was performed at various times after infection as noted in the figure legends, and adherent neutrophils were quantified as previously described (16). The vasculature was stained with PECAM-1 antibody, and neutrophils were stained with PE-conjugated anti-mouse Ly6G antibody. The number of adherent neutrophils was determined manually offline during video playback analysis using Volocity software (version 6.0.1). A neutrophil was considered to be adherent if it remained stationary for at least 30 s, and total neutrophil adhesion was quantified as the number of adherent cells within a 100-μm length of unbranched venule (25 to 40 μm in diameter) in 5 min.

**(iv) Cytokine rescue assay.** To investigate possible rescue of the various adhesin mutants by MCP-1, *Cd1d^−/−^* mice received 5 μg/25-g mouse of MCP-1 by i.v. injection 1 h prior to injection of a GFP-expressing B. burgdorferi strain (3 × 10^8^ spirochetes per mouse). The mice were infected 3 h before imaging. The total number of the GFP-expressing spirochetes outside the vasculature was counted as described in the “Vascular transmigration assay” section above.
