# Supplementary material for: Choreography of Lyme Disease Spirochete Adhesins To Promote Vascular Escape
Source: Microbiol Spectr. 2023 May 31;11(4):e01254-23. doi: 10.1128/spectrum.01254-23 (PMC10434219; doi:10.1128/spectrum.01254-23)
Supplement: Supplemental file 3 — Table S1, Fig. S1 and S2, and descriptions of Videos S1 and S2. Download spectrum.01254-23-s0001.pdf, PDF file, 0.4 MB [file spectrum.01254-23-s0001.pdf]

Supplementary Materials

Table S1. Bacterial strains

GCB strain	Description	Missing plasmids	Source/ Reference
726	B31 5A4 NP1 (*kan*) (1) + pTM61 *gent, gfp*		(2)
776	B31 5A4 NP1 (*kan*) (1) + pTM201 *gent, Tomato*		(3)
847	B31-A3 (4) + pTM61 *gent, gfp,*<br>clone 23	cp9	(5)
2958	B31 5A4		(6)
3003	B31-A3 K04 C3-14 + $p66^{D205A,D207A}$ *gent,* restored to chromosome clone 2-30 + pTM61-*strep, gfp*, clone 2-1	cp9	(5)
4458	B31-A3 Δ*ospC* + pTM61*gent, gfp,* $ospC_{B31}$	lp28-4, lp56, cp9	(7)
4452	B31-A3 *ΔospC* + pTM61*gent, gfp,* $ospC_{B31\text{-}ECM}$	cp9	(7)
4433	B31 5A4 (6) *ΔdbpA/B (gent)*<br>[pNP3 (8) from *E. coli* strain GCE1062 used to transform B31 5A4]	cp9	This work
4032	B31 5A4 *ΔdbpA/B (gent)* +pTM61*kan, gfp*<br>[pTM61*kan, gfp* (2) from *E. coli* strain GCE3951 used to transform GCB4433]	cp9, lp21	This work
4446	B31 5A4 + pTM61*kan, gfp*<br>[pTM61*kan, gfp* (2) from *E. coli* strain GCE3951 used to transform B31 5A4 (6)]		This work

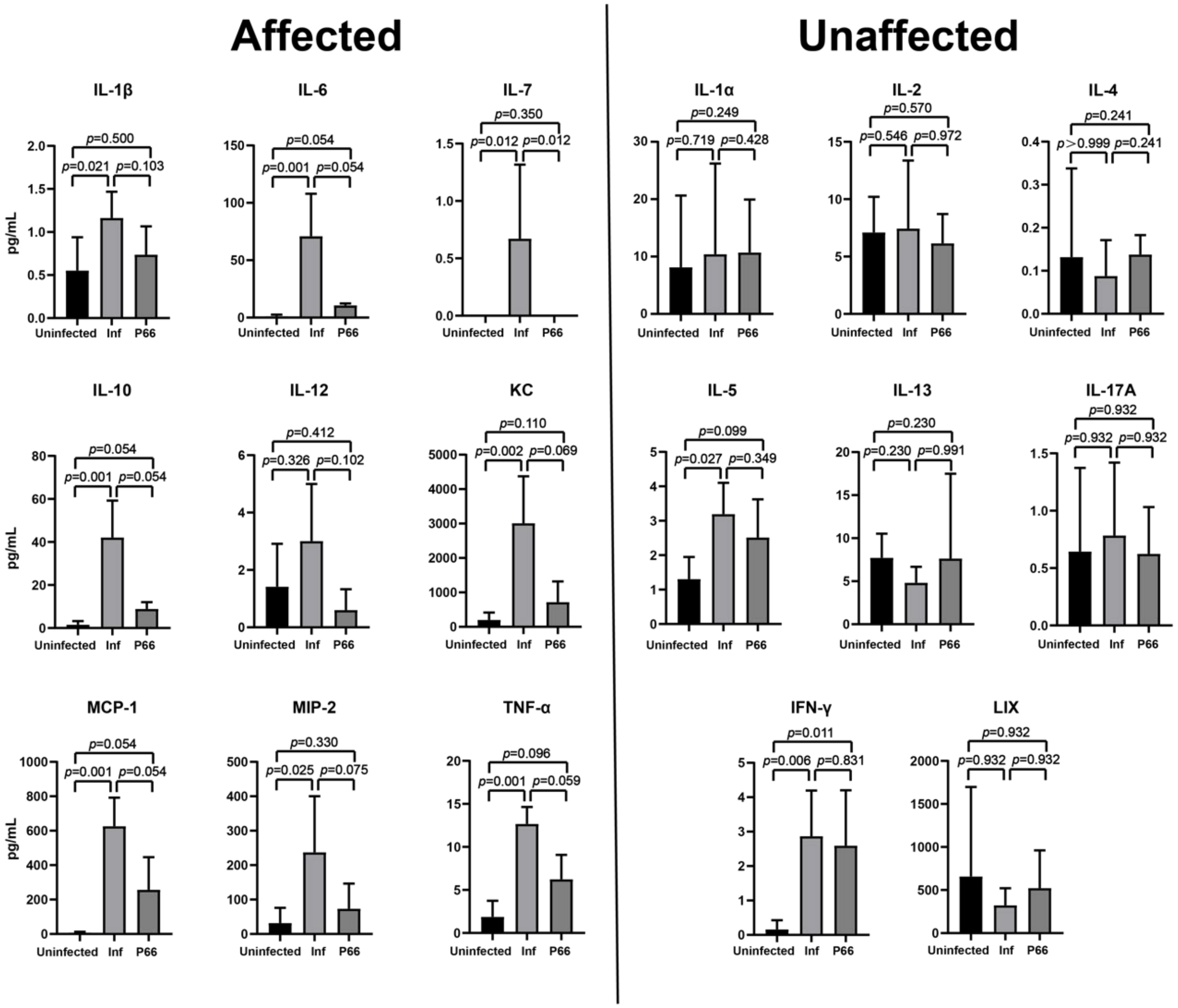

Fig. S1. Levels of 17 cytokines in uninfected, wild-type infected and P66$_{D205A,D207A}$ infected *Cd1d*$^{-/-}$ mice. Levels of 17 cytokines in three groups of mice were monitored at 24 hours post-infection. Plasma was prepared from uninfected control mice (PBS injected, n=4), wild type infected (GCB726, $3x10^8$ spirochetes by iv, n=5) and mice infected with a P66 integrin-binding mutant (P66$_{D205A,D207A}$, GCB4036, $3x10^8$ spirochetes by iv, n=5). Cytokine levels in plasma were determined using a mouse high sensitivity T-cell discovery array 18-plex. Statistical analysis was performed by ANOVA using the Kruskal-Wallis test followed by the two-stage step-up method of Benjamini, Krieger and Yekutieli.

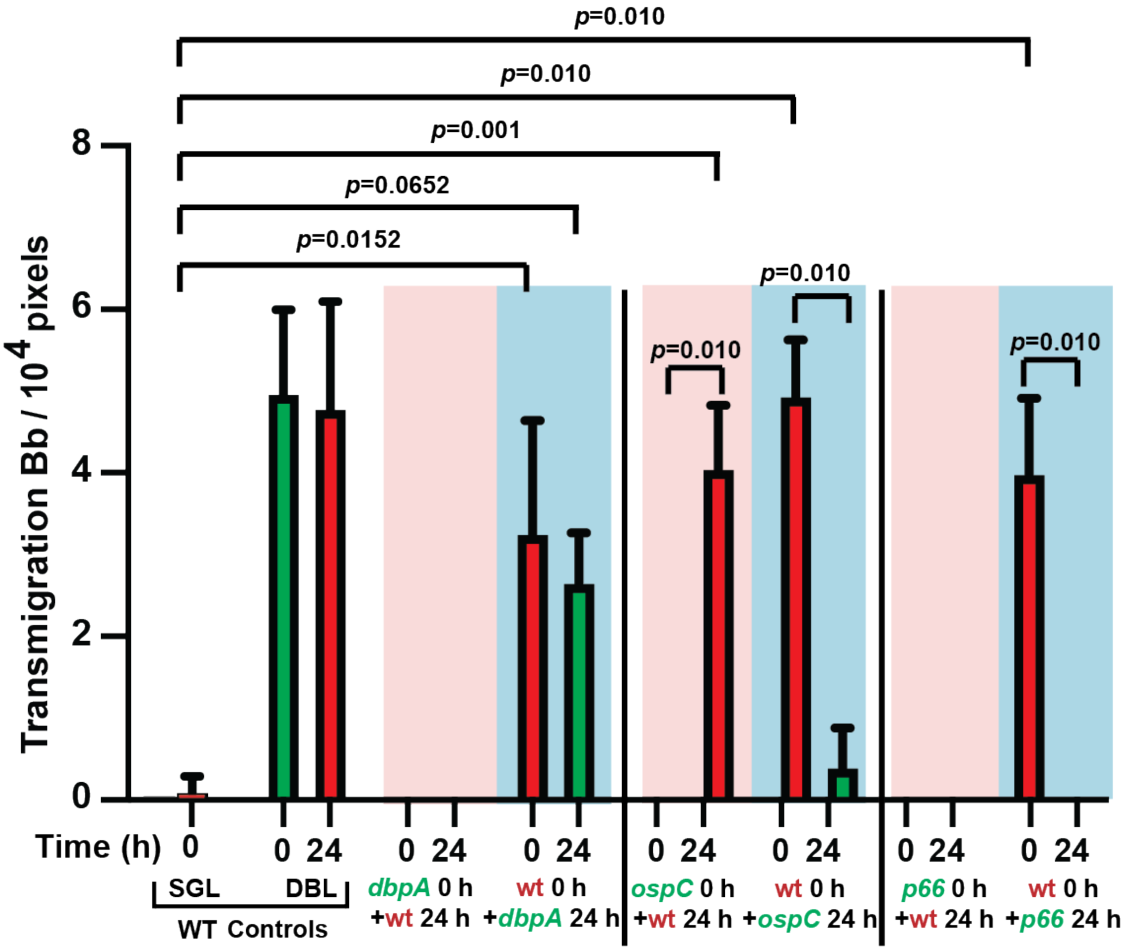

Fig. S2. Sequential double infection experiment using three adhesin mutants. The single infection (SGL) WT control group mice were injected with red spirochetes at 3 hours before imaging (n=6). The double infection (DBL) control group mice were infected with Tomato-expressing wild type spirochetes at 27 hours before imaging and GFP-expressing wild type spirochetes 24 hours later at 3 hours before imaging (n=6). The experimental group mice were infected as described in **Fig. 5**. The total number of the GFP- or Tomato-expressing spirochetes outside of the vasculature were counted; the data are plotted as the number of transmigrated spirochetes observed in an area of 10^4 pixels. Statistical significance was analyzed using the Kruskal-Wallis test followed by the two-stage step-up method of Benjamini, Krieger and Yekutieli. Error bars indicate SD. Non-significant *p* values are not shown.

Video S1. Endothelial activation in the knee joint microvasculature (uninfected mouse). Video S1 shows imaging of an uninfected mouse with the peripheral knee joint vasculature (blue) labelled with Alexa Fluor 647-conjugated anti-PECAM1 antibody and the neutrophils (red) labelled with PE-conjugated anti-Ly6G antibody. Scale bar = 50 μm. The elapsed time of each video is about 24 seconds. The video was acquired at eight frames per second.

Video S2. Endothelial activation in the knee joint microvasculature (infected mouse). Mouse infection was performed using GFP-expressing *B. burgdorferi* (GCB726; green) and video footage acquired at 24 hours post-infection. Neutrophils were stained with PE-conjugated anti-Ly6G (red) and the peripheral knee joint vasculature was stained with Alexa Fluor 647-conjugated anti-PECAM-1 (blue). Scale bar = 50 μm. The elapsed time of each video is about 24 seconds. The video was acquired at eight frames per second.

References

1. Kawabata H, Norris SJ, Watanabe H. 2004. BBE02 disruption mutants of *Borrelia burgdorferi* B31 have a highly transformable, infectious phenotype. Infect Immun 72:7147-54.
2. Moriarty TJ, Norman MU, Colarusso P, Bankhead T, Kubes P, Chaconas G. 2008. Real-time high resolution 3D imaging of the lyme disease spirochete adhering to and escaping from the vasculature of a living host. PLoS Pathog 4:e1000090.
3. Lee WY, Moriarty TJ, Wong CH, Zhou H, Strieter RM, van Rooijen N, Chaconas G, Kubes P. 2010. An intravascular immune response to *Borrelia burgdorferi* involves Kupffer cells and iNKT cells. Nat Immunol 11:295-302.
4. Elias AF, Stewart PE, Grimm D, Caimano MJ, Eggers CH, Tilly K, Bono JL, Akins DR, Radolf JD, Schwan TG, Rosa P. 2002. Clonal Polymorphism of *Borrelia burgdorferi* Strain B31 MI: Implications for Mutagenesis in an Infectious Strain Background. Infect Immun 70:2139-2150.
5. Kumar D, Ristow LC, Shi M, Mukherjee P, Caine JA, Lee WY, Kubes P, Coburn J, Chaconas G. 2015. Intravital Imaging of Vascular Transmigration by the Lyme Spirochete: Requirement for the Integrin Binding Residues of the *B. burgdorferi* P66 Protein. PLoS Pathog 11:e1005333.
6. Purser JE, Norris SJ. 2000. Correlation between plasmid content and infectivity in *Borrelia burgdorferi*. Proc Natl Acad Sci U S A 97:13865-70.
7. Lin YP, Tan X, Caine JA, Castellanos M, Chaconas G, Coburn J, Leong JM. 2020. Strain-specific joint invasion and colonization by Lyme disease spirochetes is promoted by outer surface protein C. PLoS Pathog 16:e1008516.
8. Weening EH, Parveen N, Trzeciakowski JP, Leong JM, Hook M, Skare JT. 2008. *Borrelia burgdorferi* lacking DbpBA exhibits an early survival defect during experimental infection. Infect Immun 76:5694-705.
